# Supplementary material for: Polymorphic mobile element insertions contribute to gene expression and alternative splicing in human tissues
Source: Genome Biol. 2020 Jul 27;21:185. doi: 10.1186/s13059-020-02101-4 (PMC7385971; doi:10.1186/s13059-020-02101-4)
Supplement: Supplementary file 1 — Additional file 1: Figure S1. Overview of high confidence pMEIs in GTEx individuals. Figure S2. Enrichment of pMEIs around Transcription Starting Sites (TSSs) of genes. Figure S3. Agreement of the effect direction between a pair of tissues for eQTLs (lower-left) and sQTLs (upper-right). [file 13059_2020_2101_MOESM1_ESM.pdf]

Fig. S1

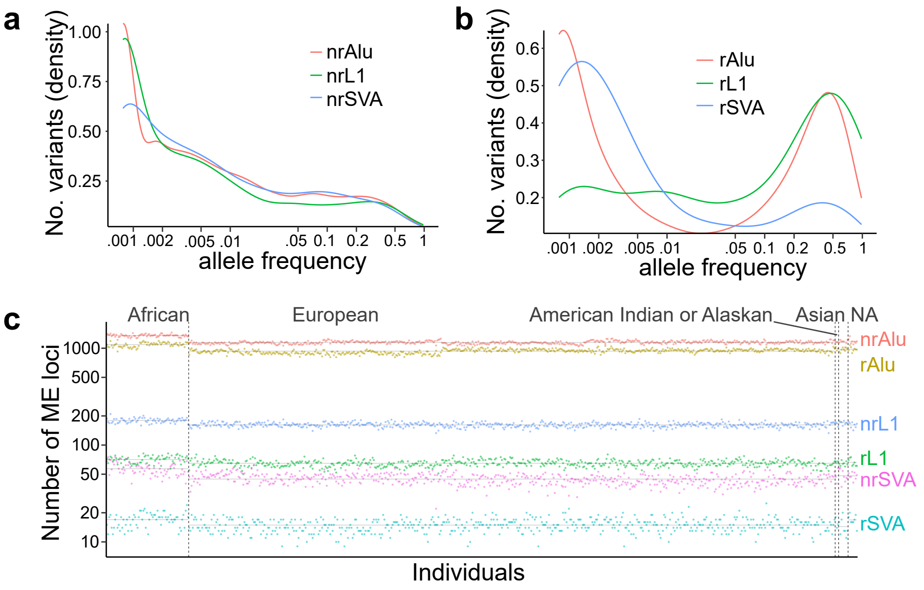

Fig. S1 Overview of high confidence pMEIs in GTEx individuals. (a, b) Allele frequency distribution of nrMEIs (a) and rMEIs (b). (c) Counts of nrMEIs (nrAlu, nrL1, nrSVA) and rMEIs (rAlu, rL1, rSVA) relative to the reference genome (GRCh38) in each individual. Individuals are grouped based on ethnic groups. NA, ethnic group unavailable.

Fig. S2

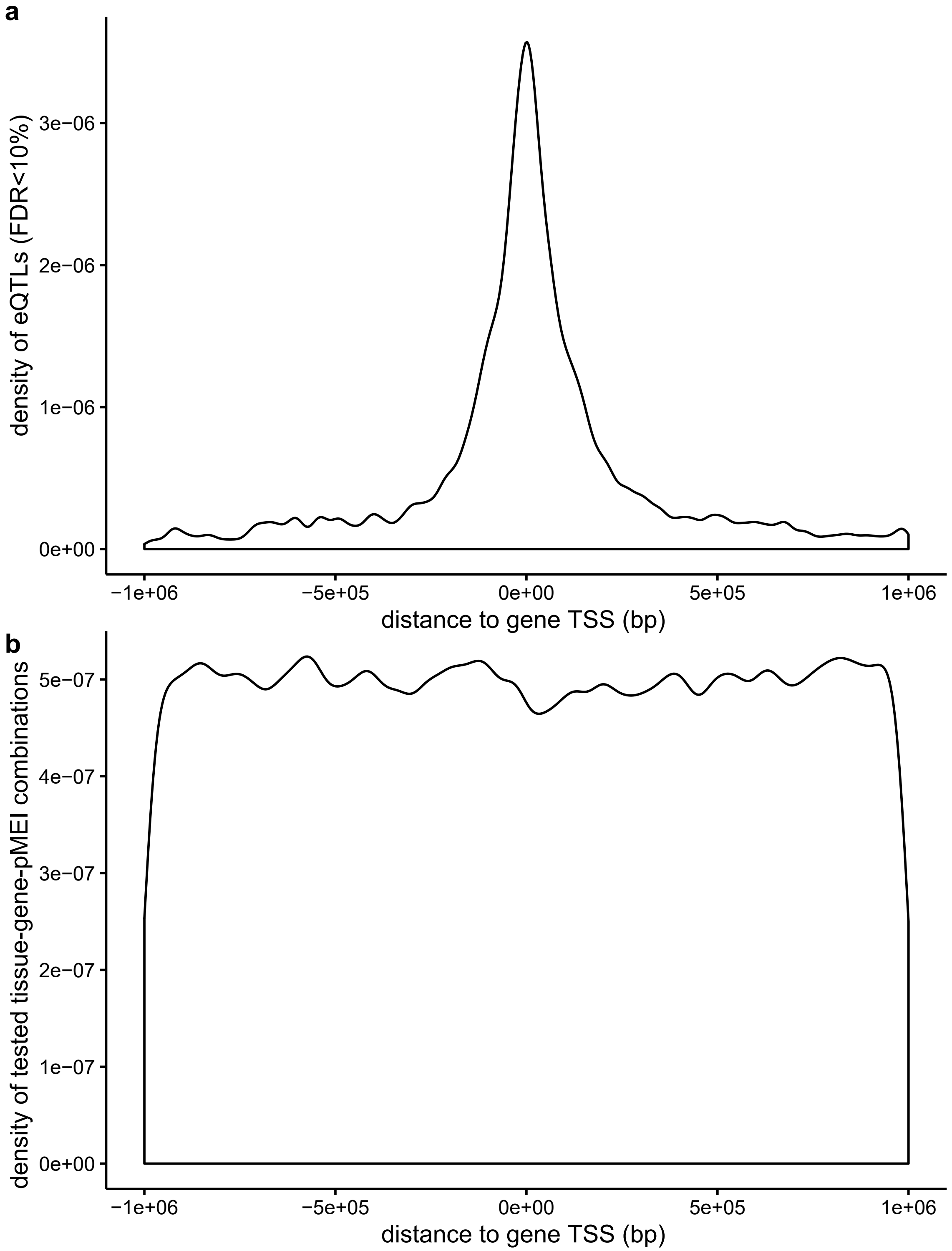

Fig. S2 Enrichment of pMEIs around Transcription Starting Sites (TSSs) of genes. (a) Density of pMEIs associated with eQTLs (tissue-gene-pMEI combinations with FDR < 0.1) around TSSs. (b) Density of pMEIs in all possible tissue-gene-pMEI combinations examined by Matrix eQTL around TSSs.

Fig. S3

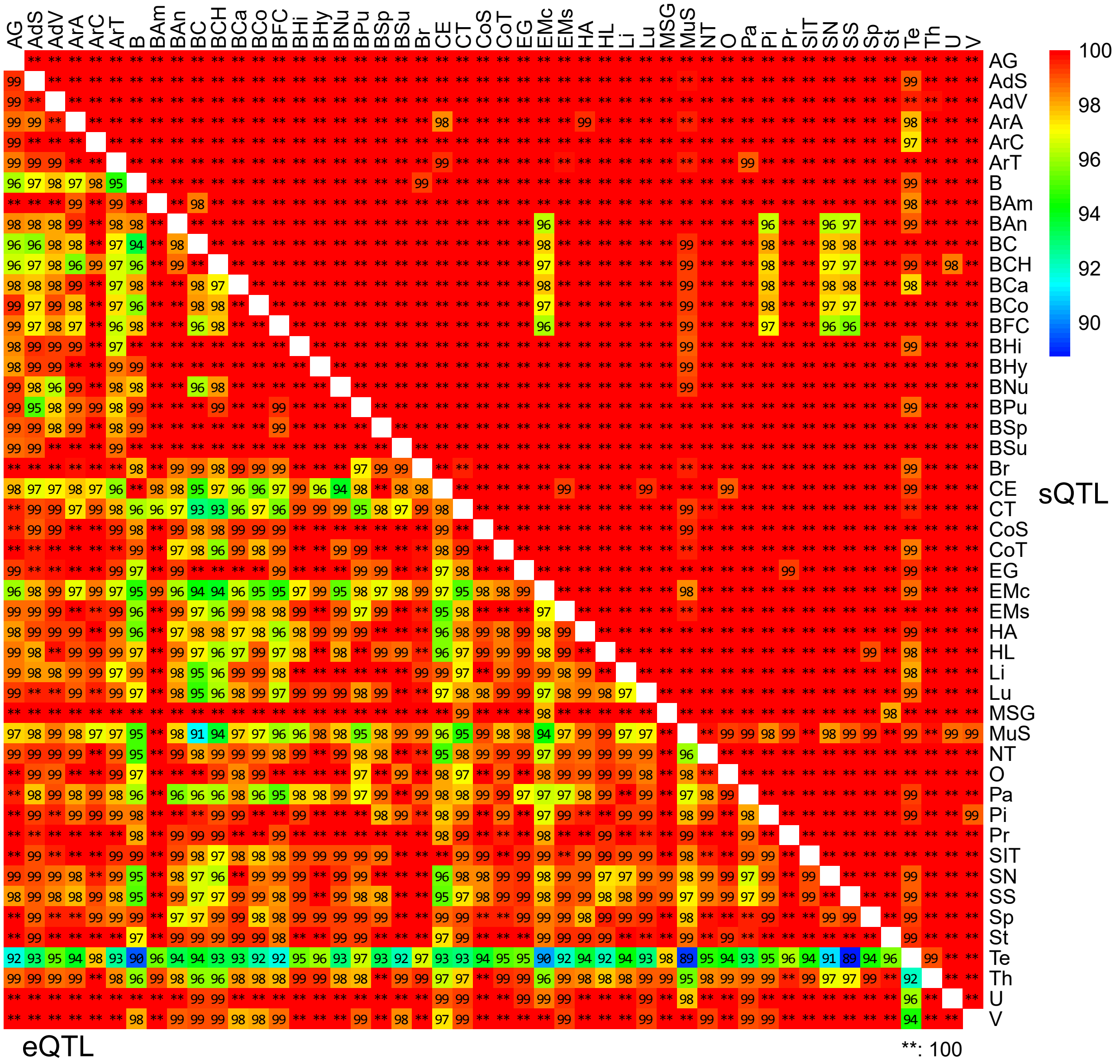

Fig. S3 Agreement of the effect direction between a pair of tissues for eQTLs (lower-left) and sQTLs (upper-right). Numbers are the percentage of shared eQTL/sQTL of two tissues with the same impact direction (the sign of beta value). Tissue abbreviations were the same as in Fig. 1. **=100% shared.
