# Supplementary material for: Polymorphic mobile element insertions contribute to gene expression and alternative splicing in human tissues
Source: Genome Biol. 2020 Jul 27;21:185. doi: 10.1186/s13059-020-02101-4 (PMC7385971; doi:10.1186/s13059-020-02101-4)
Supplement: Supplementary file 9 — Additional file 9. Review history. [file 13059_2020_2101_MOESM9_ESM.docx]

Review History

**First round of review**

**Reviewer 1**

**Are you able to assess all statistics in the manuscript, including the appropriateness of statistical tests used?**

Yes, and I have assessed the statistics in my report.

**Comments to author:**

SUMMARY

This project provides insights into an understudied area of human variation, polymorphic mobile elements. Using the unusually rich GTEx resource, they identify pMEIs, test for evidence they have functional consequences, and validate a subset using an independent assay. These results hopefully encourage others to consider pMEIs when searching for the causes of differential gene expression and splicing across tissues, individuals, and disease states.

Normally, one would hope for a replication analysis, but we recognize that there are few if any resources offering the same types of data across the same tissue types with a similar age/sex/ancestry profile. Instead, the authors experimentally evaluate whether the effects seen in the GTEx data are evident in reporter assays and cell culture, which ends up providing robust support for the evaluated loci.

The authors do a number of clever things through the paper, such as briefly introducing MEIs to the reader for context, highlighting the expected frequency of MEIs observed in the human reference sequence vs. those not observed in the reference but detected in a larger sample, the number of pMEIs observed in individuals varies by ancestry, fine-mapping eQTLs, noting the influence of sample size and noise, and so on. Overall, this is a thoughtful and careful study.

MINOR REVISIONS/QUESTIONS

1. Please review for minor spelling/grammatical errors.

2. Structural variant callers have a reputation for having poor sensitivity and specificity, although recent advances have made significant improvements in this area. Consider adding a sentence or two commenting on the specificity/sensitivity of the MELT approach used in this paper. For example, about 3/4 candidate nrMEIs and rMEIs were excluded from further analysis after QC in this text. This would help with interpretation in the discussion, where the authors note the increased number of events detected with their approach applied to a larger data set vs. prior literature.

3. Lines 83-86: To be clear, when defining the nonreference MEIs, a given MEI had to be observed in one or more GTEx samples and not the human reference, not all the GTEx samples, correct? Similarly for the reference MEIs?

4. Why use an FDR threshold of 10% rather than 5% for most analyses? Similarly, in the experimental validation section, the authors report significance using different p-values. This should be harmonized.

5. Why choose 293T cells for the reporter assay? Does this have anything to do with the tissues that appeared to have differential expression/splicing caused by eQTLs/sQTLs?


===
Please consider the following questions:
- Are the methods appropriate to the aims of the study, are they well described, and are necessary controls included? If not, please specify what is required.
Yes, with minor questions described above.
- Are the conclusions adequately supported by the data shown? If not, please explain
Yes
- Are sufficient details provided to allow replication and comparison with related analyses that may have been performed? If not, please specify what is required.
Yes
- Does the work represent a significant advance over previously published studies?
Yes
- Is the paper of broad interest to others in the field, or of outstanding interest to a broad audience of biologists?
Yes

**Reviewer 2**

**Are you able to assess all statistics in the manuscript, including the appropriateness of statistical tests used?**

No, I do not feel adequately qualified to assess the statistics

**Comments to author:**

This is a very well-written manuscript that attacks a topic that is sorely in need of attention. I have only a few relatively minor criticisms.

Lines 148-151: The authors note that there is no statistical differences when comparing the types of TEs and their impacts and refer to Figure 1e. I agree with that assessment for L1 and Alu but a quick look at SVAs and noncoding genes suggests otherwise. It would be good to have the numbers for these tests for the reader to see.

Lines 161-163: I'm curious as to whether there is a statistical association between SNPs and the pMEIs. In other words, are they linked? If so, was there any attempt to disentangle the possible impacts on expression outcomes?

Line 182: change 'casual' to 'causal'.

Line 189: This header is very oddly worded.

Line 208: Change to "may also be"

Line 279: Remove 'that'

Line 299: Change 'difference' to 'different'

Line 332: Change to 'validated'
===
Please consider the following questions:
- Are the methods appropriate to the aims of the study, are they well described, and are necessary controls included? If not, please specify what is required.
Yes.
- Are the conclusions adequately supported by the data shown? If not, please explain
Yes.
- Are sufficient details provided to allow replication and comparison with related analyses that may have been performed? If not, please specify what is required.
Yes.
- Does the work represent a significant advance over previously published studies?
Yes.
- Is the paper of broad interest to others in the field, or of outstanding interest to a broad audience of biologists?
Yes.

**Authors Response**

**Point-by-point responses to the reviewers’ comments:**

Comment

*Response*

Reviewer #1

1. Please review for minor spelling/grammatical errors.

*We further reviewed the manuscript and corrected several spelling/grammatical errors. We also asked a native speaker to review the manuscript and revised several sentences to improve the clarity.*

2. Structural variant callers have a reputation for having poor sensitivity and specificity, although recent advances have made significant improvements in this area. Consider adding a sentence or two commenting on the specificity/sensitivity of the MELT approach used in this paper. For example, about 3/4 candidate nrMEIs and rMEIs were excluded from further analysis after QC in this text. This would help with interpretation in the discussion, where the authors note the increased number of events detected with their approach applied to a larger data set vs. prior literature.

*Thanks for the suggestion. We added additional comments regarding the differences in MEI-caller’s performance in the discussion. The section (starting from line 309) now reads:*

*“A previous study showed that different analysis methods can produce very different eQTL results, even with the same raw dataset [28]. It is also known that different ME identification programs have different sensitivity and specificity. In addition, the pMEI selection can further introduce discrepancies among studies [42]. For example, our analyses focused on a small set of common pMEIs, which accounts for only 17% of the high-quality call set. To assess the consistency of our eQTL analysis with other studies, we compared the eQTLs identified in LCLs with an eQTL study of LCLs from 1KGP samples [26].”*

3. Lines 83-86: To be clear, when defining the nonreference MEIs, a given MEI had to be observed in one or more GTEx samples and not the human reference, not all the GTEx samples, correct? Similarly for the reference MEIs?

*The reviewer is correct. The MEIs we identified are polymorphic among GTEx samples, none of the insertions are present in all GTEx samples. More specifically, nonreference MEIs are MEIs that are not present in the reference human genome but detected in at least one GTEx sample; reference MEIs are MEIs that are present in the human reference genome and absent in at least one GTEx sample.*

4. Why use an FDR threshold of 10% rather than 5% for most analyses? Similarly, in the experimental validation section, the authors report significance using different p-values. This should be harmonized.

*We thank the reviewer for the comment. It is true that 5% FDR is a common cutoff when conducting multiple comparisons. Here, as the number of pMEIs tested are relatively small, we chose a less stringent cutoff of 10% FDR to maximize the QTL discovery. Because we performed fine-mapping analysis in the following step, we expect the false-positive loci will not be identified as the causal variants for the QTL loci. This is also the cutoff used in the GTEx study of the impact of structural variants on gene expression (Chiang et al, 2017).*

*We apologize for the confusion of the different p-values reported in the experiment validation section. The values were intended to report the p-value ranges, not the p-value cutoffs. We revised the section and used “p < 0.05” in both eQTL and sQTL sections for consistency.*

*Reference:*

*Chiang C, Scott AJ, Davis JR, Tsang EK, Li X, Kim Y, Hadzic T, Damani FN, Ganel L, Consortium GT, et al: The impact of structural variation on human gene expression. Nat Genet 2017, 49:692-699.*

5. Why choose 293T cells for the reporter assay? Does this have anything to do with the tissues that appeared to have differential expression/splicing caused by eQTLs/sQTLs?

*Because all of the pMEIs that we tested were detected as eQTLs or sQTLs in multiple tissues, these loci likely represent strong eQTLs or sQTLs that would be likely to affect gene expression in multiple cell lines as well. We choose to use 293T because they are easy to transfect and are frequently used in ectopic reporter assays, even in cases where the likely biological function is in another cell type. We added a sentence in the methods section expanding on our selection of 293T cells. The sentence (line 489) now reads:*

*“**293T cells were selected because they are easy to transfect and are frequently used in ectopic reporter assays.”*

Reviewer #2

Lines 148-151: The authors note that there is no statistical differences when comparing the types of TEs and their impacts and refer to Figure 1e. I agree with that assessment for L1 and Alu but a quick look at SVAs and noncoding genes suggests otherwise. It would be good to have the numbers for these tests for the reader to see.

*We appreciate the that the reviewers performed the analysis to validate the conclusions. We did perform the statistical analysis with data from Figure 1e, and there is significant difference for SVAs and noncoding genes (p-value 2e-79 for the binomial test). However, this result is based on all eQTLs (i.e., all pMEI-gene-tissue combinations). Some pMEIs are significant eQTLs in multiple tissues and/or affect multiple genes, and they can potentially bias the results, especially for small dataset, such as the SVAs. To control for this issue, we selected a single best QTL for each pMEI for the testing, and the analysis on unique pMEIs yielded no significant difference. We now added a brief description of the analysis using all eQTLs (as seen in Fig. 1e) and the selection process for the analysis described in the main text.*

*As the reviewer suggested, we also added a supplemental table (Table S2) that contains the counts and p-values of each category for both analyses. The section (starting line 149) now reads:*

*“To determine if the presence/absence of an ME has a directional impact on the gene expression, we examined the association between the direction of the gene expression change (positive or negative beta value) with the presence or absence of an ME. We observed several significant differences (e.g., SVA for non-coding genes, Fig. 1e, Table S2). However, because some pMEIs are eQTLs in multiple tissues and/or affect multiple genes, these pMEIs can potentially bias the result, especially for small dataset such as SVAs. To control for this bias, we selected a single best eQTL for each pMEI for the testing. Using one eQTL per pMEI locus, we observed no statistically significant difference in the direction of the effect for any of the three types of pMEIs (Alu, L1, SVA), for either coding or non-coding genes (Table S2).*

Lines 161-163: I'm curious as to whether there is a statistical association between SNPs and the pMEIs. In other words, are they linked? If so, was there any attempt to disentangle the possible impacts on expression outcomes?

*The reviewer is correct that there are linkages between SNPs and pMEIs. In the fine-mapping step, we used the program CAVIAR to disentangle the causal contribution of SNPs and pMEIs based on the linkage disequilibrium among them and their evidence of eQTL. As described in the “Fine-mapping causal pMEIs for eGenes” section, pMEIs were identified as one of the potential causal variants for 13.98% of eGenes. For the remaining loci, nearby SNPs showed stronger association with the gene expression variation.*

Line 182: change 'casual' to 'causal'.

*Thank you for pointing out this typo. We corrected as suggested.*

Line 189: This header is very oddly worded.

*We changed the header from “Identify pMEIs-associated sQTLs” to “Identification of pMEI-associated sQTLs”. For line 104, we also changed the title from “Identify pMEI-associated eQTLs” to “Identification pMEI-associated eQTLs”.*

Line 208: Change to "may also be"

*We changed the text as suggested.*

Line 279: Remove 'that'

*We changed “showed that” to “predict”.*

Line 299: Change 'difference' to 'different'

*We changed the text as suggested.*

Line 332: Change to 'validated'
*We changed the text as suggested.*

**Second round of review**

**Reviewer 1**

Thank you to the authors for responding so completely to my questions and suggestions.

**Reviewer 2**

I find that the authors addressed my concerns well and that the manuscript is now ready for publication.
